# Analysis options for high-throughput sequencing in miRNA expression profiling

**DOI:** 10.1186/1756-0500-7-144

**Published:** 2014-03-13

**Authors:** Tomasz Stokowy, Markus Eszlinger, Michał Świerniak, Krzysztof Fujarewicz, Barbara Jarząb, Ralf Paschke, Knut Krohn

**Affiliations:** 1 Systems Engineering Group, Institute of Automatic Control, Silesian University of Technology, Gliwice, Poland; 2 Nuclear Medicine and Endocrine Oncology Department, Maria Sklodowska-Curie Memorial Cancer Center and Institute of Oncology, Gliwice, Poland; 3 Division of Endocrinology and Nephrology, University of Leipzig, Leipzig, Germany; 4 Interdisciplinary Center for Clinical Research (IZKF), University of Leipzig, Liebigstr. 21, 04103 Leipzig, Germany; 5 Genomic Medicine, Department of General, Transplant, and Liver Surgery, Medical University of Warsaw, Warsaw, Poland

**Keywords:** High-throughput sequencing, Follicular thyroid cancer, miRNA expression, Microarrays

## Abstract

**Background:**

Recently high-throughput sequencing (HTS) using next generation sequencing techniques became useful in digital gene expression profiling.

Our study introduces analysis options for HTS data based on mapping to miRBase or counting and grouping of identical sequence reads. Those approaches allow a hypothesis free detection of miRNA differential expression.

**Methods:**

We compare our results to microarray and qPCR data from one set of RNA samples. We use Illumina platforms for microarray analysis and miRNA sequencing of 20 samples from benign follicular thyroid adenoma and malignant follicular thyroid carcinoma. Furthermore, we use three strategies for HTS data analysis to evaluate miRNA biomarkers for malignant versus benign follicular thyroid tumors.

**Results:**

High correlation of qPCR and HTS data was observed for the proposed analysis methods. However, qPCR is limited in the differential detection of miRNA isoforms. Moreover, we illustrate a much broader dynamic range of HTS compared to microarrays for small RNA studies. Finally, our data confirm hsa-miR-197-3p, hsa-miR-221-3p, hsa-miR-222-3p and both hsa-miR-144-3p and hsa-miR-144-5p as potential follicular thyroid cancer biomarkers.

**Conclusions:**

Compared to microarrays HTS provides a global profile of miRNA expression with higher specificity and in more detail. Summarizing of HTS reads as isoform groups (analysis pipeline B) or according to functional criteria (seed analysis pipeline C), which better correlates to results of qPCR are promising new options for HTS analysis. Finally, data opens future miRNA research perspectives for HTS and indicates that qPCR might be limited in validating HTS data in detail.

## Background

Two decades ago miRNAs - small ribonucleic acids about 22 nucleotides in length - have entered the scientific stage and since then became recognized as important regulators of gene expression at the molecular level [1-3]. While binding to mRNA they decide the fate of their target by either affecting the half-life of these nucleic acids or changing the translational success of the transcript. These essential functions made their expression profiles an increasingly well studied readout in many biological processes and diseases [4-6]. miRNAs function in an one-to-many transcripts relationship and potentially affect gene expression networks which makes them important pathobiological markers for disease diagnosis including cancer [7-9].

Recently high-throughput sequencing (HTS) using next generation sequencing (NGS) techniques became useful in digital gene expression profiling [10,11]. Short read length and high coverage are especially suited for counting miRNA prevalence and calculating differential expression. Compared to qPCR, the accepted gold standard in transcript quantification, HTS offers a genome wide approach and allows overcoming the limitations of array based analysis which is restricted to miRNA molecules provided by databases and suffers from cross-detection prone hybridization methods.

So far, studies comparing the three methods are rare and their results cannot be directly compared. Some of them challenge the quality of qPCR in miRNA expression measurements [12], other show good qPCR – microarray [13-15] or HTS – microarray [16-19] concordance.

Apart from other studies our work for the first time compares all three methods in one set of RNA samples from follicular thyroid tumors. In the molecular diagnosis of these tumors it is most important to find reliable markers that allow an accurate classification of histologically [20] and, more important cytologically [21] inconclusive samples. Although both HTS and microarray data are likely to define differentially expressed miRNAs as potential biomarker candidates it is essential that their differential expression is reproducible with qPCR which is the preferable method for routine molecular diagnosis.

In our study we use Illumina platforms for microarray analysis and miRNA sequencing of samples from benign follicular adenoma (FA) and malignant follicular thyroid carcinoma (FTC). Our sequencing analysis applies current algorithms for polishing, parsing and aligning short reads to the human genome (hg19) followed by intersecting miR coordinates [22-26] and an adaption to the sequences spotted on the microarrays followed by classical statistical analysis of data. Moreover, we introduce an analysis option that is not based on mapping and allows hypothesis free and de novo detection of differentially expressed miRNA isoforms. We also correlate the output of HTS data analysis pipelines to microarray and qPCR results. Finally, miRNAs differentially expressed between the two types of tumor were compared to miRNA markers proposed in papers which previously described FTC – FA differences [27-29].

## Methods

### Thyroid tumor samples

10 follicular thyroid carcinoma (FTC) and 10 follicular thyroid adenoma (FA) samples were selected for miRNA profiling by HTS. Hematoxylin and eosin (HE) stained histology sections of all tumor samples were independently rated by two experienced pathologists to guarantee the precise tumor classification. Approval of the ethics commission of the medical faculty of the University of Leipzig and written consent of the respective patients was obtained.

### RNA isolation and library preparation

Total RNA was extracted from 20-50 mg snap-frozen thyroid tissue using 1 mL Trizol reagent. Reverse transcription was done with miScript Reverse Transcription kit (Qiagen, Hilden, Germany) according to the manufacturer’s protocols in a 10 μL reaction using 1 μg of total RNA. 500 ng of total RNA prepared from the tumor samples was used in the small RNA protocol with the TruSeq™ Small RNA sample prepkit v2 (Illumina) according to the instructions of the manufacturer. The barcoded libraries were size restricted between 140 and 165 bp, purified and quantified using the Library Quantification Kit - Illumina/Universal (KAPA Biosystems) according to the instructions of the manufacturer.

### High-throughput sequencing

A pool of ten libraries was used for cluster generation at a concentration of 10 nM using an Illumina cBot. Sequencing of 50 bp was performed with an Illumina HighScan-SQ sequencer using version 3 chemistry and flowcell according to the instructions of the manufacturer.

### High-throughput sequencing data analysis

For naming of the sequences the following nomenclature in concordance with miRBase 18 was used. According to miRBase the entry sequence represents the most frequent isoform of a miRNA. A hairpin miRNA or pre-miRNA sequence is the precursor of up to 2 canonical mature miRNA sequences (denoted as 5p or 3p). Isoforms of such a miRNA sequence vary around the miRBase entry with a certain offset at the 5′ end or 3′ end or a slight difference in nucleotide sequence (miRNA point mutations and SNPs).

**Figure 1:**
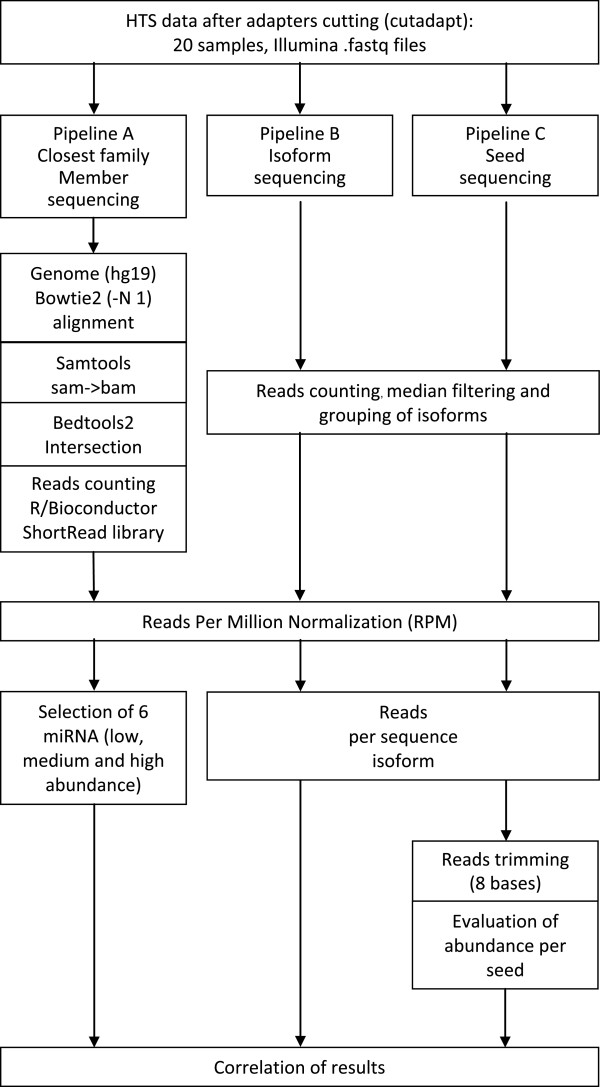
**Data analysis pipelines of high-throughput sequencing.** Three different analysis approaches were applied to small RNA profiling in samples from follicular thyroid tumors. Results were obtained from mapping reads to the human genome (hg19) – closest family member sequencing (Pipeline A), mapping independent isoform sequencing (Pipeline B) and seed analysis (Pipeline C). The closest family member sequencing workflow (Pipeline A) uses bowtie2 mapping to the human genome obtained from the public repository. The isoform sequencing pipeline returns the frequency of each individual sequence isoform. Seed analysis merges counts of individual isoforms with the same miRNA seed sequence and therefore targets biological importance of miRNA isoform families (sequences) which could potentially act in concert as regulators of thyroid cells.

High-throughput sequencing data was analyzed according to the three pipelines presented in Figure 1. The aim of the different analysis pipelines is to measure the expression of miRNA with different approaches: the classical approach - alignment of reads, and two new approaches - counting of isoforms which is independent of any database entries and seed analysis which focuses analysis to the bases that are most likely functionally relevant.

At first, we removed adapters sequences from raw reads (50 bases, fastq files) of all 20 samples (http://www.ncbi.nlm.nih.gov/geo/query/acc.cgi?acc=GSE39112) using Cutadapt software [30]. From the remaining sequences only those 15–27 bases long were kept for further analysis because they most likely contain mature miRNA sequences. Sequences with median quality lower than phred = 30 were discarded from the analysis. To monitor the success of the adapter cutting and filtering we used the FastQC program (Bioinformatics Group at the Babraham Institute, UK).

At this point further analysis was split into pathways: (i) mapping based pipeline A intersecting known miRNAs coordinates from miRBase after alignment to the human genome and (ii) pipeline B and C which count identical reads first and then annotate individual reads as miRNA isoform when they contain a miRBase entry sequence or map to hairpin miRNA sequences. Moreover sequences that did not succeed in this annotation step were annotated as uncharacterized small RNA sequences if they align to the human genome. Pipelines B and C aim for de novo analysis of thyroid tumor miRNA and small RNA profiles and therefore include all sequences independent of a homology to known miRNA molecules.

The first pipeline is based on bowtie2 alignment to the sequence of the human genome (hg19) allowing one mismatch and alignments to multiple reference targets. All other parameters were set as default. Data were compressed with Samtools view [31] to bam format followed by intersecting human genomic miRNA coordinates from miRbase (hsa.gff3) using BEDTools [26]. Reads were counted using the R/Bioconductor programming environment [32] by application of the ShortRead library [33]. All counts were normalized by Reads Per Million [34], described by the formula:

(1) $$\mathrm{RPM}=R_{\mathrm{miR}}/R_{\mathrm{all}}*10^{6}$$

where:

R_miR_ – number of reads mapped to a particular miRNA reference in the sample,

R_all_ – total number of reads mapped in the sample,

RPM – normalized miRNA abundance value.

Out of the available normalization methods including DESeq [35] and TMM [36] we have chosen RPM which is a simple method that has been frequently used in current miRNA studies [37,38]. Moreover, we normalized our data with DESeq and compared these results to RPM normalization (Additional file 1).

**Figure 2:**
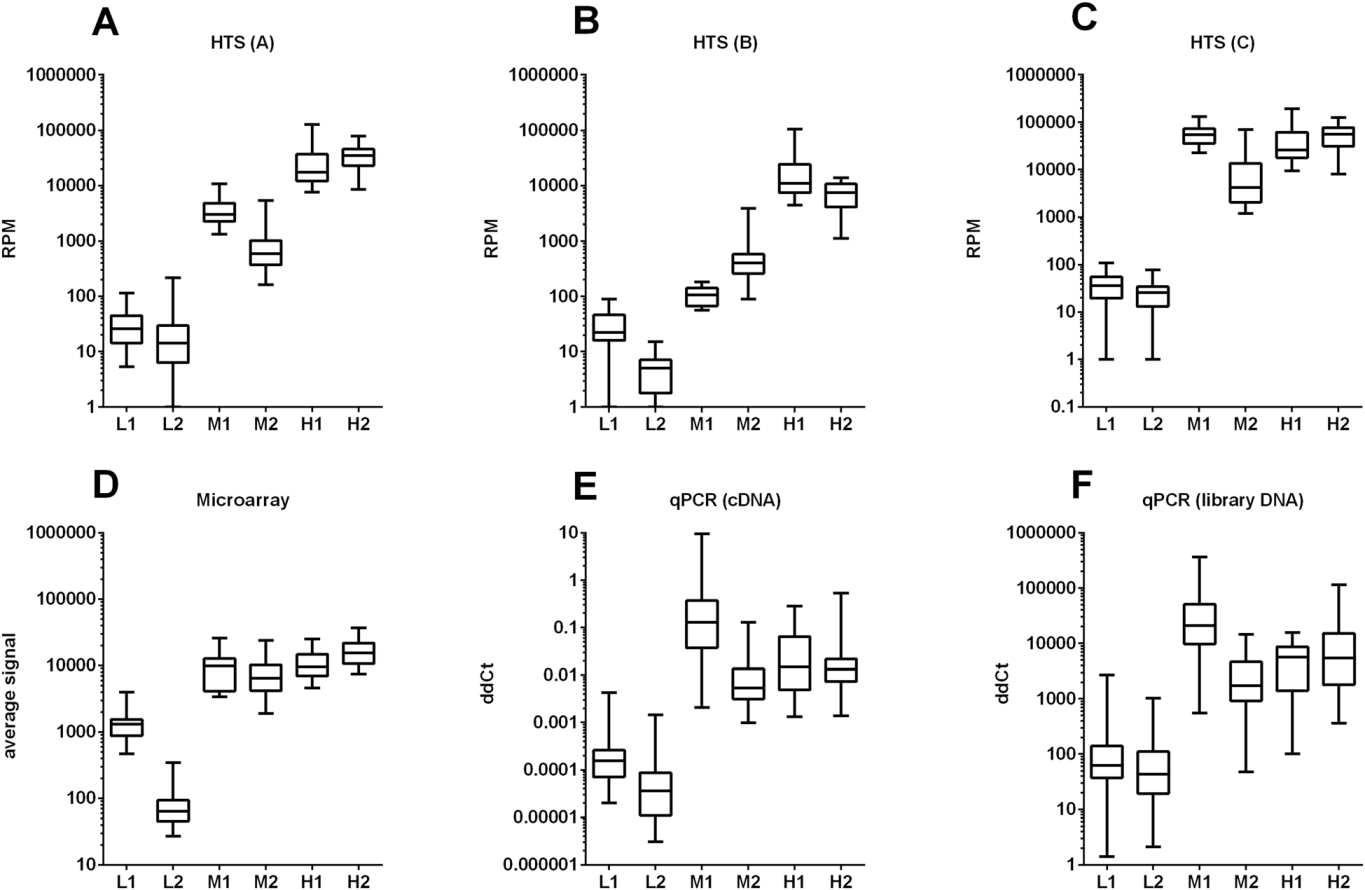
**qPCR validation of miRNA profiling using HTS and microarray data.** Profiling of 6 miRNAs using microarray analysis, qPCR and results of three different HTS analysis pipelines **(A-C)** shows a very similar expression pattern. Box plots present distribution of appropriately normalized miRNA expression in 20 samples. The seed sequence for pipeline C is derived from the miRBase entry sequence of the respective miRNA. Best concordance of miRNA abundance is seen between microarray data **(D)**, qPCR from cDNA **(E)**, qPCR from sequencing library DNA **(F)** and HTS data analysis pipeline C **(C)**. miRNAs were labeled according to the key: L1 – hsa-miR-27b-5p, L2 - hsa-miR-708-3p, M1 – hsa-miR-30e-5p, M2 – hsa-miR-146a-5p, H1 – hsa-miR-181a-5p, H2 – hsa-miR-143-3p.

6 miRNA sequences representing low, medium and high abundance in HTS data analysis pipeline A were used for correlation with microarray, qPCR and HTS data from analysis pipelines B and C (Tables 1 and 2, Figure 2). Low and high abundance miRNAs were selected from the lower and upper quartile of expression, respectively. Medium abundance miRNAs stem from the interquartile range. Differences between abundance levels are significant as presented in the Table 1 and Figure 2A.

**Table 1:** Canonical mature miRNAs selected for qPCR validation

**miRNA**	**HTS (A)**		**qPCR target sequence**
	**Abundance class**	**RPM**	
hsa-miR-27b-5p	Low1 (L1)	33	agagcuuagcugauuggugaac
hsa-miR-708-3p	Low2 (L2)	26	caacuagacugugagcuucuag
hsa-miR-30e-5p	Medium1 (M1)	3639	uguaaacauccuugacuggaag
hsa-miR-146a-5p	Medium2 (M2)	1002	ugagaacugaauuccauggguu
hsa-miR-181a-5p	High1 (H1)	28011	aacauucaacgcugucggugagu
hsa-miR-143-3p	High2 (H2)	36537	ugagaugaagcacuguagcuc

**Table 2:** Correlation of mean miRNA expression

**qPCR (cDNA)**	***0.99	*0.90	*0.86	*0.68	**0.96
-	**qPCR (library)**	*0.87	*0.90	*0.73	***0.99
-	-	**Array-signal**	*0.87	*0.82	*0.89
-	-	-	**HTS (A)**	**0.93	**0.95
-	-	-	**-**	**HTS (B)**	*0.82
-	-	-	-	-	**HTS (C)**

*p < 0.05,

**p < 0.01,

***p < 0.001.

Pipelines B and C of HTS data analysis also start with trimmed raw sequences but count the number of reads of each small RNA isoform present in the fastq files. Their main goal is to identify isoform specific and miRNA-seed specific (functional) abundance. This counting approach allows inclusion of sequences that do not exactly match miRBase entry sequences or have an offset at the 5′ or 3′ end which could be cell type or organ specific. Moreover uncharacterized small RNAs could be detected and evaluated. For the purpose of counting a memory efficient Perl script was implemented (Sequence_counter.pl). The counting algorithm is similar to approaches proposed in the literature [35,39], however our Perl script is designated for string operations which is more time efficient in some cases. We further analyzed our data with miRDeep2 [39] to find novel miRNAs in our data set. Because this analysis did not lead to the detection of novel miRNA species we concentrated on the evaluation of miRNA isoforms obtained from our counting algorithm (pipelines B and C). Counting introduces a huge amount of read variants that might contain sequencing errors or represent sequences of unknown origin, function and small RNA classification. Therefore, we propose a median abundance filter to remove RNA artifacts from the thyroid tissue data. Only sequences detected in at least half of the 20 samples will pass this filter and were considered in downstream analysis. Counts of sequences were also normalized to RPM values assuming: 

RmiR – number of sequence counts in the sample,

Rall – total number of reads in the sample.

Pipeline B stops at this point resulting in RPM values obtained for each detected isoform. Normalized data were used for the correlation analysis of 6 miRNAs and the comparison to published FTC-FA markers.

Pipeline C continues to focus on the seed region, known as the most important regulatory part of mature miRNA [4,40,41]. We decided to summarize all miRNA isoforms that share 8 base pairs at the 5′ end of the sequence. Moreover, in most qPCR strategies these 8 base pairs are included in the smallest consensus target. The task of isoform summarization was performed using the Seed_analysis.r script in the R environment. Table 3 compares the performances in the validation of miRNA markers proposed in the literature for all three pipelines and the DESeq algorithm [35].

**Table 3:** Cross-comparison of array and high-throughput sequencing data with differential regulated miRNAs from other studies

**miRNA**	**Regulation of FTC versus FA (fold change)**					**Citation**	**Methodology**	**Samples**
	**HTS (A)**	**HTS (B)**	**HTS (C)**	**DESeq**	**Reference**			
hsa-miR-192-5p	Down (0.67)	Down (0.52)	Down (0.50)	Down (0.55)	Up (1.34)	Weber et al., JCEM [28]	Selection of markers: miRNA microarray	Selection 12 FTC & 8 FA
hsa-miR-192-3p	**-	**-	**-	**-				
hsa-miR-197-5p	**-	**-	**-	**-	Up (1.82)			
hsa-miR-197-3p	Up (1.26)	Up (1.50)	Up (1.30)	Up (1.39)			Validation: qPCR	Validation 5 FTC & 4 FA
hsa-miR-328	Down (0.67)	Down (0.59)	Down (0.59)	Down (0.62)	Up (1.49)			
hsa-miR-346	**-	**-	**-	**-	Up (1.39)			
hsa-miR-146b-5p	Up (2.26)	- (1.04)	- (1.06)	- (0.80)	Up (2.8)	Nikiforova et al., JCEM [27]	Selection of markers: qPCR assay (TaqMan human panel with 158 miRNAs)	Selection 9 FTC & 8 FA
hsa-miR-146b-3p	Up (3.14)	**-	Up (1.50)	**-				
hsa-miR-155-5p*	- (1.13)	Down (0.56)	Down (0.50)	Down (0.58)	Up (5.5)			
hsa-miR-155-3p	**-	**-	**-	**-				
hsa-miR-187-5p	**-	**-	**-	**-	Up (33.4)			
hsa-miR-187-3p	**-	**-	**-	**-				
hsa-miR-221-5p	- (0.99)	**-	- (0.84)	**-	Up (3.6)		Validation: qPCR	Validation 5 FTC & 4 FA
hsa-miR-221-3p	Up (2.01)	Up (3.08)	***Up (1.78)	Up (3.77)				
hsa-miR-222-5p	**-	**-	**-	**-	Up (4.5)			
hsa-miR-222-3p	Up (1.96)	Up (1.52)	***Up (1.78)	Up (1.77)				
hsa-miR-224-5p	Up (2.26)	**-	**-	**-	Up (6.2)			
hsa-miR-224-3p	**-	**-	**-	**-				
hsa-miR-199b-5p*	Up (1.58)	Up (1.31)	- (1.04)	Up (1.33)	Down (0.026)	Rossing et al., J MolEndocrinol. [29]	Selection of markers: miRNA microarray	Selection and validation 12 FTC & 12 FA
hsa-miR-199b-3p*	Up (1.35)	Up (1.90)	- (1.19)	- (1.20)	Down (0.082)			
hsa-miR-144-5p	Down (0.53)	Down (0.31)	Down (0.34)	Down (0.36)	Down (0.14)		Validation: leave one out cross-validation, cell lines	
hsa-miR-144-3p	Down (0.47)	Down (0.50)	Down (0.51)	Down (0.43)				

*Very high miRNA abundance of one sample causes increase of mean expression in one of the two tumor groups and may affect fold change result.

**Low abundance (median RPM < 10) or no signal detected.

***miRNAs have the same seed sequence.

### Microarray data analysis

miRNA data were obtained from the Illumina scanner in the form of normalized log2 signal intensities. Probe sequences were re-annotated with information from miRBase version 18 according to http://www.ebi.ac.uk/arrayexpress/files/A-GEOD-8179/A-GEOD-8179.adf.txt. 1145 normalized microarray intensities were used for correlation analysis and to compare dynamic ranges. Microarray data were obtained for 20 samples analyzed with HTS, without technical replicates.

### qPCR methodology

#### qPCR validation of miRNAs with low, medium and high prevalence in all HTS data samples

The qPCR validation of hsa-miR-27b (L1), hsa-miR-708 (L2), hsa-miR-30e (M1), hsa-miR-146a (M2), hsa-miR-181a (H1), and hsa-miR-143-1 (H2) was done in both, cDNA samples prepared from the original RNAs using the miScript RT kit (Qiagen, Hilden, Germany) according to the manufacturer’s instructions, and the library DNAs prepared for HTS as described above (see library preparation). qPCR was done in a 10 μL reaction using the miScript SYBR Green PCR kit (Qiagen, Hilden, Germany) and commercially available miScript Primer Assays (forward primers; Qiagen, Hilden, Germany) and the reference RNU6B in combination with the miScript Universal primer (reverse primer) for the cDNA templates [42], and in combination with a library specific reverse primer for the library DNA templates (see Additional file 2 for qPCR outline and primers). qPCR was performed on a Roche LightCycler 480 according to the kit manufacturer’s instructions. PCRs were processed through an initial denaturation at 95°C for 15 min and by 40 cycles of a 3-step PCR, including 15 sec of denaturation at 94°C, a 30 sec annealing phase at 55°C and an elongation phase at 70°C for 30 sec.

#### qPCR validation of hsa-miR-30 isoforms in HTS libraries

The qPCR validation of isoforms of the hsa-miR-30 family was done in the library DNAs prepared for HTS, which were generated as described above. qPCR was done in a 10 μL reaction using the miScript SYBR Green PCR kit (Qiagen, Hilden, Germany) and isoform specific forward and reverse primers (see Additional file 2 for qPCR outline and primers). qPCR was performed on a Roche LightCycler 480 according to the kit manufacturer’s instructions. PCRs were processed as detailed above.

## Results

### High-throughput sequencing data

We analyzed 4.7 ± 1.5 million raw sequence reads per sample (count and RPM numbers are given as mean or mean ± standard deviation) from an Illumina small RNA sequencing run using three pipelines (Figure 1). After adapter cutting 6.6*10^5 ± 4.5*10^5 reads were accepted for further analysis. Direct mapping (closest family member sequencing pipeline A) of trimmed reads to human genomic sequences using bowtie2 resulted in 9.9*10^5 ± 8.3*10^5 hits. These hits intersect with 1101 ± 153 out of 4446 genomic coordinates for miRbase entries as determined by annotation using BEDtools. Our results correspond to 91.51 ± 3.36% of all reads per sample aligned to the human genome (hg19) compared to 61.09 ± 14.45% aligned to mature miRBase sequences. Additionally, 69.23 ± 15.79% of all reads per sample mapped to hairpin miRBase sequences. The percentage of reads that comprise uncharacterized small RNA sequences is 22.3 ± 3.2%. Additional file 3 lists the ten most abundant small RNA sequences together with their genomic origin.

In isoform analysis (pipeline B) we counted individual sequence isoforms and normalized these counts to sequence abundance as reads per million (RPM). We also compared RPM to DESeq normalization (Additional file 1). Pearson correlation of data normalized with the two algorithm is 0.97. One of the 20 samples had a higher number of total reads compared to all other which resulted in a separate line in the graph (see upper diagonal line in Additional file 1). Pipeline B resulted in 18855 small, various RNA sequences that were detected in at least half of the 20 thyroid samples. Furthermore, these small sequences aligned to 502 ± 88 hairpin precursor miRBase sequences. Using miRDeep2 [39] none of the remaining uncharacterized small RNAs were identified as new miRNAs. miRBase v18 annotation was attributed to those isoform groups that contain an exact miRNA entry sequence. Seed analysis (pipeline C) merged the 18855 isoforms from pipeline B into 5189 isoform groups with identical seed sequences (i.e. the first 8 bases of each miRNA).

### Comparison of high-throughput sequencing and microarray

**Figure 3:**
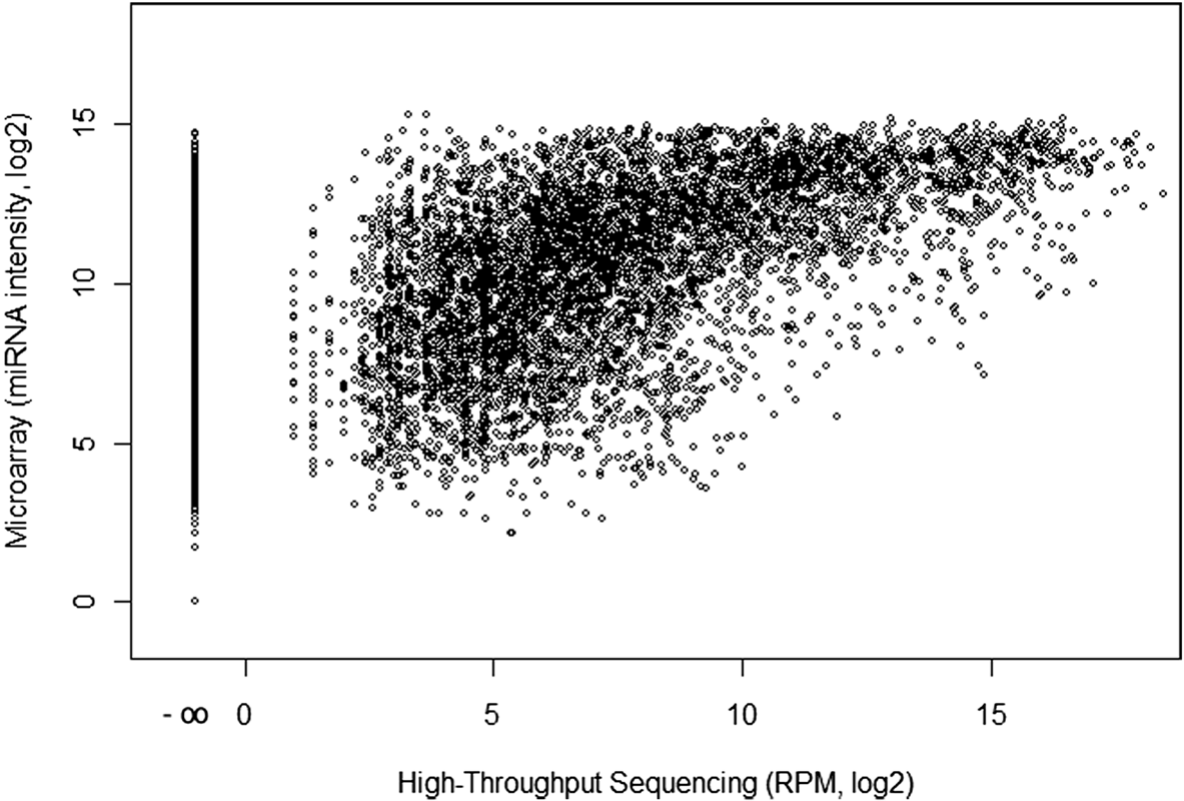
**Comparison of Illumina high-throughput sequencing and Illumina miRNA Bead Arrays v2 performance in thyroid tumor samples.** Each dot represents the abundance of one mature miRNA in 1 of 20 samples in HTS (x-axis) and the fluorescent intensity of the corresponding spot on the microarray (y-axis) of all 20 samples. Expression values are based on normalized data, assuming perfect matching of microarray probe sequence and HTS sequence reads. The figure illustrates the saturation of microarray signals compared to the number of HTS reads and a lack of specificity of microarray probe hybridizations by the presence of fluorescent signal for miRNA targets without any HTS reads (x-axis position -∞). Because RPM normalization of low read counts generates visual artifact (column-like data structures between 0 and 5 RPM) we excluded all miRNAs with a very low abundance of one or two reads in individual samples. The correlation coefficient between the microarray and HTS data set is 0.67.

First we compare average fluorescent intensities detected in microarray analysis with the number of reads mapped to all probe sequences of the miRNA expression BeadChip v2 (Illumina) as reference. This analysis follows the closest family member sequencing pipeline but uses microarray probe sequences and their annotation instead of human genome sequences. In Figure 3 we show the comparison for all 20 thyroid samples and 1145 individual sequences. We detect a strong saturation of microarray signal intensities compared to a wide dynamic range of the HTS data. Moreover, miRNA sequences that were not present in the HTS data show a wide spectrum of signal intensities on the array (see the most left column of dots in Figure 3). We also compared microarray signal intensities to the results of HTS pipeline B and C (Additional file 4). The saturation effect of the microarray is also seen in the plot for the pipeline B data (Additional file 4A). However, comparing microarray signal intensities to pipelines B and C data (Additional file 4) does not reveal a meaningful correlation.

### Validation with qPCR

Table 1 shows the 6 mature miRNAs we selected for qPCR validation based on the level of prevalence calculated with the closest family member sequencing pipeline A. This method outputs RPM normalized read numbers after alignment to human genome sequences with bowtie2 mapping software. Our selection includes miRNAs with low, medium and high prevalence in all HTS data samples and therefore covers a wide dynamic range. miRNAs were selected from groups of prevalence at random, assuming they must be abundant in all the samples. Selected miRNAs were investigated with 2 additional high-throughput sequencing analysis approaches (pipelines B and C) using the set of 20 samples. Figure 2 summarizes analysis of HTS with pipelines A-C (2A-C), microarray (2D) and qPCR (2E and F). As seen in the direct comparison of microarray and HTS data, the dynamic range of RPM is wider than the array signal intensities. This is most prominent for the medium and high prevalence sequences (Figure 2, M1-H2). A comparison of HTS data (pipeline A, closest family member sequencing, Figure 2A) with qPCR using cDNA reverse transcribed from total RNA (Figure 2E) shows a very good concordance for 5 out of 6 miRNAs.

### Correlation of small RNA qPCR and high-throughput sequencing data

To compare HTS analysis pipelines with experimental data from microarray and qPCR data we calculated Pearson correlation for all three miRNA expression profiling techniques. To detect the miRNA levels with the lowest experimental noise (which might appear e.g., due to different RT efficiencies), we used qPCR not only with the cDNAs prepared from the original RNA samples, but also with the HTS library DNAs as targets. Table 2 gives the correlation coefficients of the pair wise comparisons between methods for the expression of the selected miRNAs in 20 samples (Table 1). We observed a good correlation between the qPCR methods and microarray data with a slightly better coefficient for qPCR validation starting from reverse transcribed total RNA (r = 0.90, p < 0.05). The correlation of qPCR and HTS data strongly depends on the analysis pipeline. For both qPCR methods (cDNA and library DNA) seed analysis pipeline C (r = 0.96 and 0.99) performs much better than the closest family member pipeline A (r = 0.86 and 0.90). Overall, the highest correlation for the HTS analysis with pipeline C is detected in the comparison to qPCR validation of library DNA (r = 0.99, p < 0.001). qPCR validation starting from reverse transcribed total RNA with seed analysis pipeline C data resulted in a very similar coefficient (r = 0.96, p < 0.01). In general, qPCR and high-throughput sequencing data are highly correlated when the appropriate analysis method (seed analysis pipeline C) is applied.

### Limits of qPCR validation

**Figure 4:**
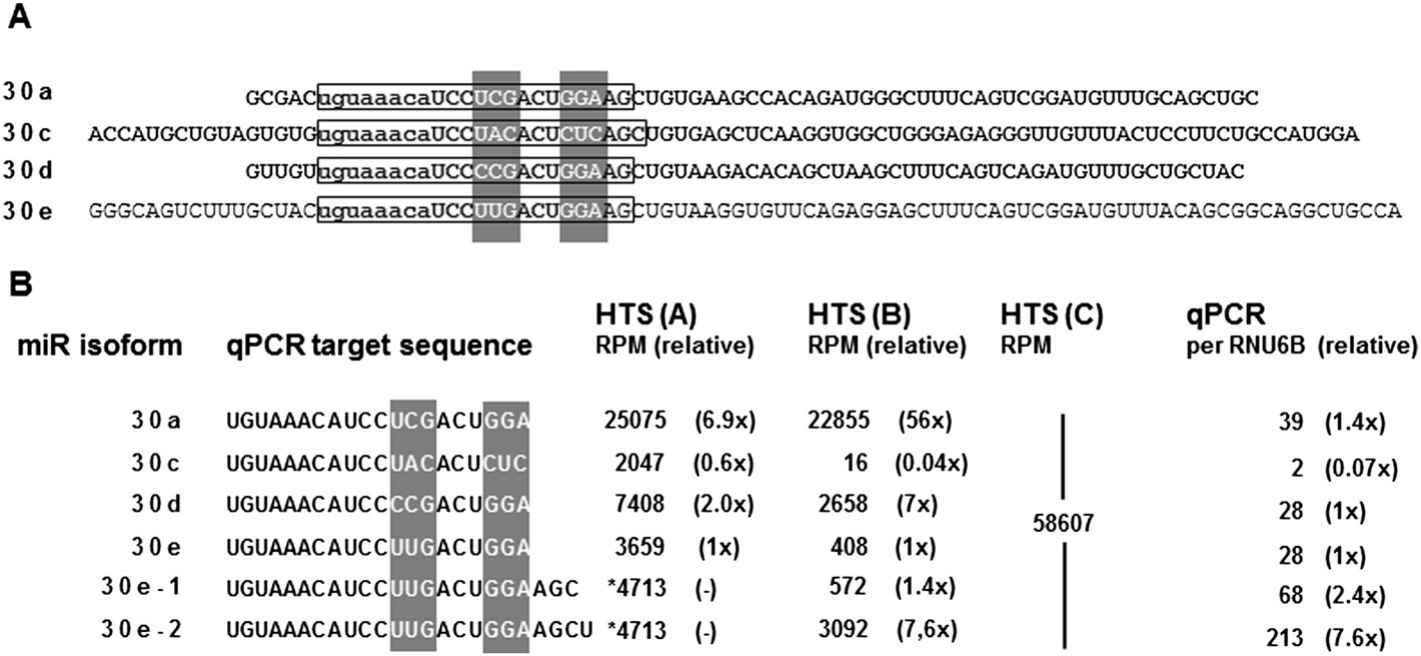
**Isoforms of the hsa-miR-30 family.** The hsa-miR-30 family comprises of a large number of overlapping mature miRNA sequences that might interfere with expression profiling. Mapping HTS reads to the human genome (pipeline A) detects 4 significantly overlapping sequences shown as hairpin sequences in **A**. Open boxes mark the sequences of mature 5′ miRNAs that were further validated. The sequence part that allows differentiation between the family members is marked in grey. The seed sequence common to all members is shown in lower case. **(B)** Currently, only high-throughput sequencing can clearly differentiate between hsa-miR-30 family members, additionally providing a chance to evaluate expression of hsa-miR-30e isoforms – 30e-1 and 30e-2. Means of miRNA expression in 20 samples using HTS data and qPCR for hsa-miR-30e-5p and interfering sequences (i.e. 30a, c and d) are listed. Although using specific forward and reverse primers for the detection of isoforms of the hsa-miR-30 family, qPCR that targets specific hsa-miR-30 family members clearly lacks specificity to detect 1 base changes in the target sequence. *- HTS pipeline A detection of the hairpin sequence for has-miR-30e. **- Pipeline C generates a sum of all isoforms with the same seed sequence.

qPCR from cDNA template resulted in unexpected high expression values for hsa-miR-30e (Figure 2E-M1) compared to HTS analysis with pipeline A (3A). Strikingly, DNA from the sequencing libraries (Figure 2F) as template for qPCR led to results identical to cDNA. Therefore, the differences for hsa-miR-30e were not explained by the different templates, which rules out a methodological bias of the library preparation. These differences are better explained by the highly diverse isoform distribution (Figure 4) of hsa-miR-30e-5p (30e) and sequence similarities to hsa-miR-30a-5p (30a), 30c-5p (30c) and 30d-5p (30d).

Isoform sequencing pipeline B detects a total of 466 isoforms which share the same seed sequence but contain offsets at both the 5′ and the 3′ end and a high number of substitutions. The seed sequence (first 8 bases) of these isoforms matches miRBase entry sequences for different miRNAs of the hsa-miR-30 family. In principal, 30a, 30d and 30e are more related since the attribution of an individual isoform to either of these miRNAs could be based on two single nucleotide mismatches (C versus U) in the miRBase entry sequences. The distance between 30a, 30d and 30e to 30c is a little higher (4 bases). Very likely qPCR based on the target sequence for 30e will at least also partially amplify targets from 30a and 30d. Concordance of qPCR results with seed analysis pipeline C which summarizes reads from members of the hsa-miR-30 family with the same seed sequence will therefore be higher.

### Evaluation of published miRNA FTC-FA markers

Table 3 summarizes sequencing results for 22 canonical mature 5p or 3p miRs listed in miRBase which originate from 12 hairpin pre-miRNA transcripts recently published as FTC – FA markers [27-29]. We tested their differential expression in our sample set of 10 FTC and 10 FA. Our HTS analysis pipelines detect 11 to 14 of the 22 miRNAs, depending on the applied analysis pipeline. When we consider a fold-change of at least 1.25 as increase/up-regulation and 0.8 as decrease/down-regulation our analysis allows a call in 9 to 12 of the 22 miRNAs (Table 3). Calls from the three HTS pipelines are concordant for 7 miRNAs. Best concordance of sequencing with published data is seen for the human hairpin miRNAs 144, 146, 197, 221 and 222. For hsa-miR-144 both 5p and 3p sequences were confirmed as differentially expressed. This result corroborates to DESeq - the most frequently used RNA-Seq expression profiling algorithm [35].

In contrast to previously published markers, the 6 miRNAs evaluated in this study do not show any significant differences between the classes of malignity as presented in the Additional file 5.

### miRNA expression profiling guidelines

In a summary of the results we propose a guidance for miRNA expression profiling techniques. For experiments with aim of differential expression measurements we propose the high-throughput sequencing analysis pipeline based on seed analysis. This method will grant a functional importance of findings and the highest likelihood of qPCR validation success. In case of clinical studies, applied to samples with small cytological differences the best approach is to apply the isoform sequencing analysis pipeline. Such a method will grant the most sensitive differentiation option and most accurate use of HTS data. It also allows de novo detection of differentially expressed isoforms. Finally, for experiments with known, significant biological differences between sample classes cost of analysis per sample is an issue so microarray techniques will still be an acceptable solution. It is important to underline that among many microarray platforms only those with the newest annotations (miRBase > 17) should be considered.

## Discussion

Here we present data for miRNA expression profiling using high-throughput sequencing. Our study introduces analysis options for HTS data based on mapping to miRBase or counting and grouping of identical sequence reads without mapping. In general, direct mapping (pipeline A) results in a miRNA expression profile that allows referencing to existing knowledge including the genomic origin of the miRNA. Pipeline B generates a hypothesis free expression profile that might be most useful in the differential diagnosis of disease. And finally, pipeline C implements a potential functional target of miRNAs by focusing to the seed sequence. Furthermore, we compare HTS results to microarray data and quantitative PCR from the same sample set of 20 thyroid tumors. Our first analytic strategy (pipeline A) follows the established algorithms and is focused on mapping to the human genome. The two other strategies start with grouping of identical sequence reads after trimming based on full length sequence or just 8 bases known as miRNA seed sequence. Differential expression is then calculated for each sequence group. Groups are labeled with the homologous miRBase entry or miRNA family. Finally, we test the usefulness of three different pipelines for the evaluation of follicular thyroid tumor markers and compare the results with microarray and qPCR data.

Comparison of HTS and microarray data clearly illustrates the disadvantages of microarray analysis: the lack of probe specificity and significant saturation of signal. Direct comparison reveals that some miRNA microarray probes that result in a fluorescent signal on the array were not found in the HTS reads from the same samples. Among the ten probe sequences with the highest signal intensity but without HTS reads (Additional file 6) we detect a number of long G/C stretches that might cause cross hybridization. Moreover, many sequences poorly represented in HTS data show an array signal both in raw and in normalized microarray data. This is most likely also explained by frequent cross hybridization during array processing, which is typical for the detection of short targets because higher stringency washes would also risk the loss of the specific signal.

Nevertheless, the overall correlation of microarray analysis and HTS observed in our study is satisfactory. Therefore, microarrays provide a global profile of miRNA expression with relatively small experimental costs that might lack detail and specificity but is useful for the detection of differential expression.

A close validation of 6 miRNAs leads to the conclusion that all three investigated platforms can be cross-compared and their data correlate to a high extent. Multiplatform miRNA expression summary shows the best concordance of microarray, qPCR and HTS seed analysis (pipeline C) patterns, which is also reflected in higher exact Pearson correlation values. The sum of isoforms with the same seed sequence calculated by pipeline C most likely better represents the expression of a heterogeneous family like hsa-miR-30 although it is not able to discriminate the expression of individual family members. However, in the light of the functional importance of the seed sequence [4,40,43] this analysis might be of additional significance. Direct mapping to the database sequences (pipeline A) or counting only the isoform with the exact database entry sequence for miR-30e (pipeline B) might be the most sequence specific analysis but very likely substantially underestimates the expression and functional relevance of the mature hsa-miR-30 family sequences. Moreover, our data from qPCR analysis of the hsa-miR-30 family members indicate that qPCR might be limited in validating HTS data in detail. The qPCR signal with primers targeted to hsa-miR-30e is identical to amplification of hsa-miR-30d and only slightly lower (about 40%) than amplification of hsa-miR-30a. In contrast, mapped reads are detected in a ratio of 1:2:7, respectively. This suggests that most of the qPCR signal with primers targeting hsa-miR-30e comes from amplification of hsa-miR-30a, which implies that this qPCR approach at least lacks specificity to detect 1 base changes in the target sequence.

Thanks to the high accuracy of HTS reads, it is possible to evaluate miRNA abundance on the basis of individual sequence isoforms, although it is often not easy to unambiguously annotate a certain isoform with a miRBase entry. We show an example of isoform ambiguity for the hsa-miR-30 family where the database entry sequence for the mature form of hsa-miR-30e differs by a single nucleotide from hsa-miR-30a and by two nucleotides from hsa-miR-30d. In addition, hsa-miR-30c is also closely related with just 2 to 3 mismatches. Moreover, hsa-miR-30c and hsa-miR-7, which have been reported as papillary thyroid carcinoma biomarkers [44] cover isoforms originating from different genome locations. In such cases differentiation of miRNA isoforms is still difficult and requires further genome wide studies. However, differential expression based on groups of individual sequence isoforms allows de novo detection of potentially important small RNAs without previous knowledge or changing database annotation.

miRNAs previously described in the literature as follicular thyroid cancer biomarkers [27-29] do not present isoform ambiguity, which could cause uncertainty of HTS data evaluation. So all three HTS analysis strategies confirmed hsa-miR-197-3p, hsa-miR-221-3p, hsa-miR-222-3p, and both 3p and 5pof hsa-miR-144 as cancer biomarkers in our sample set (Table 3). In contrast, miRNAs used for technical evaluation are not differentially expressed between FTC and FA, as shown in Additional file 5.

## Conclusion

High-throughput sequencing added a powerful tool for miRNA profiling. The field is rapidly moving and new possibilities offered by HTS should be considered in any study. Future studies need to clarify whether the expression profile of certain isoforms is of functional or diagnostic relevance. Alternatively, it is possible that summarizing of HTS reads by functional criteria (seed analysis pipeline C), which better correlates to results of qPCR and microarray is more adequate. Our data opens perspectives for future research but still many HTS data aspects remain to be solved.

### Availability and implementation

Additional files 7, 8 and 9 provide raw counts for analysis pipelines A, B and C, respectively.

Data access for publication:

http://www.ncbi.nlm.nih.gov/geo/query/acc.cgi?acc=GSE39112.

Software implemented for the purposes of publication: http://www.zmnieo.home.pl/autoinstalator/joomla/index.php/bioinformatics.html.

## Competing interests

The authors declare that they have no competing interests.

## Authors’ contributions

Experiment design: TS, BJ, RP, KK, Samples acquisition: ME, BJ, RP, Biological experiments: ME, KK, Data analysis: TS, MŚ, KF, Data submission to repository: MŚ, Manuscript preparations: TS, ME, MŚ, KK, Manuscript revisions: KF, BJ, RP. All authors accepted the final version of the manuscript. All authors read and approved the final manuscript.

## Supplementary Material

Additional file 1 **The figure shows the comparison of DESeq and RPM normalization of Illumina high-throughput sequencing data.** Each dot represents the RPM normalized (x-axis) and DESeq normalized (y-axis) HTS miRNA isoform data (pipeline B) for 1 of 20 thyroid cancer HTS data samples. Pearson correlation of data normalized independently with 2 normalizations is equal to 0.97. Click here for file

Additional file 2 The table provides the qPCR outline with the respective primers. Click here for file

Additional file 3 The table lists abundant uncharacterized small RNA sequences and gives the respective genomic origin. Click here for file

Additional file 4 **The figure shows the comparison of Illumina high-throughput sequencing and Illumina miRNA Bead Arrays v2 performance in thyroid tumor samples.** Each dot represents the sum of abundance of all individual sequence isoforms generated by pipeline B that contains the sequence of one array probe **(A)** or the sum of abundance of all individual sequence isoforms generated by pipeline C that share the same seed sequence **(B)** in 1 of 20 samples in HTS (x-axis) and the fluorescent intensity of the corresponding spot **(A)** or aggregation of the fluorescent intensity of corresponding spots with the same seed sequence **(B)** on the microarray (y-axis) of all 20 samples. Expression values are based on normalized data, assuming perfect matching of microarray probe sequence and HTS sequence reads **(A)** or the same seed sequence **(B)**. The figures illustrate low correlation of microarray signals and the number of HTS reads and a lack of specificity of microarray probe hybridizations by the presence of fluorescent signal for miRNA targets without any HTS reads (x-axis position -∞). The Pearson correlation coefficient between the microarray and the HTS pipeline B or C data sets is −0.012 and 0.040, respectively. Click here for file

Additional file 5 **The figure shows the expression of 6 selected miRNAs separated by tumor malignancy condition.** Consecutive rows illustrate pipelines A, B and C, while box plot axis RPM normalized and log2 transformed miRNA abundance. No significant differential miRNA expression between tumor classes is observed in studied analysis pipelines. Click here for file

Additional file 6 The table lists microarray probe sequences without HTS reads but high fluorescent intensity. Click here for file

Additional file 7 The table provides raw counts for miR-30a-5p, −30c-5p, −30d-5p and -30e-5p as well as for hairpin sequence of miRNA-30e (pipeline A). Click here for file

Additional file 8 The spreadsheet provides raw counts for all individual reads (pipeline B). Click here for file

Additional file 9 The spreadsheet provides raw counts of all seed family members (pipeline C). Click here for file
