# Supplementary figures and images for: Analysis options for high-throughput sequencing in miRNA expression profiling
Source: BMC Res Notes. 2014 Mar 13;7:144. doi: 10.1186/1756-0500-7-144 (PMC4007773; doi:10.1186/1756-0500-7-144)

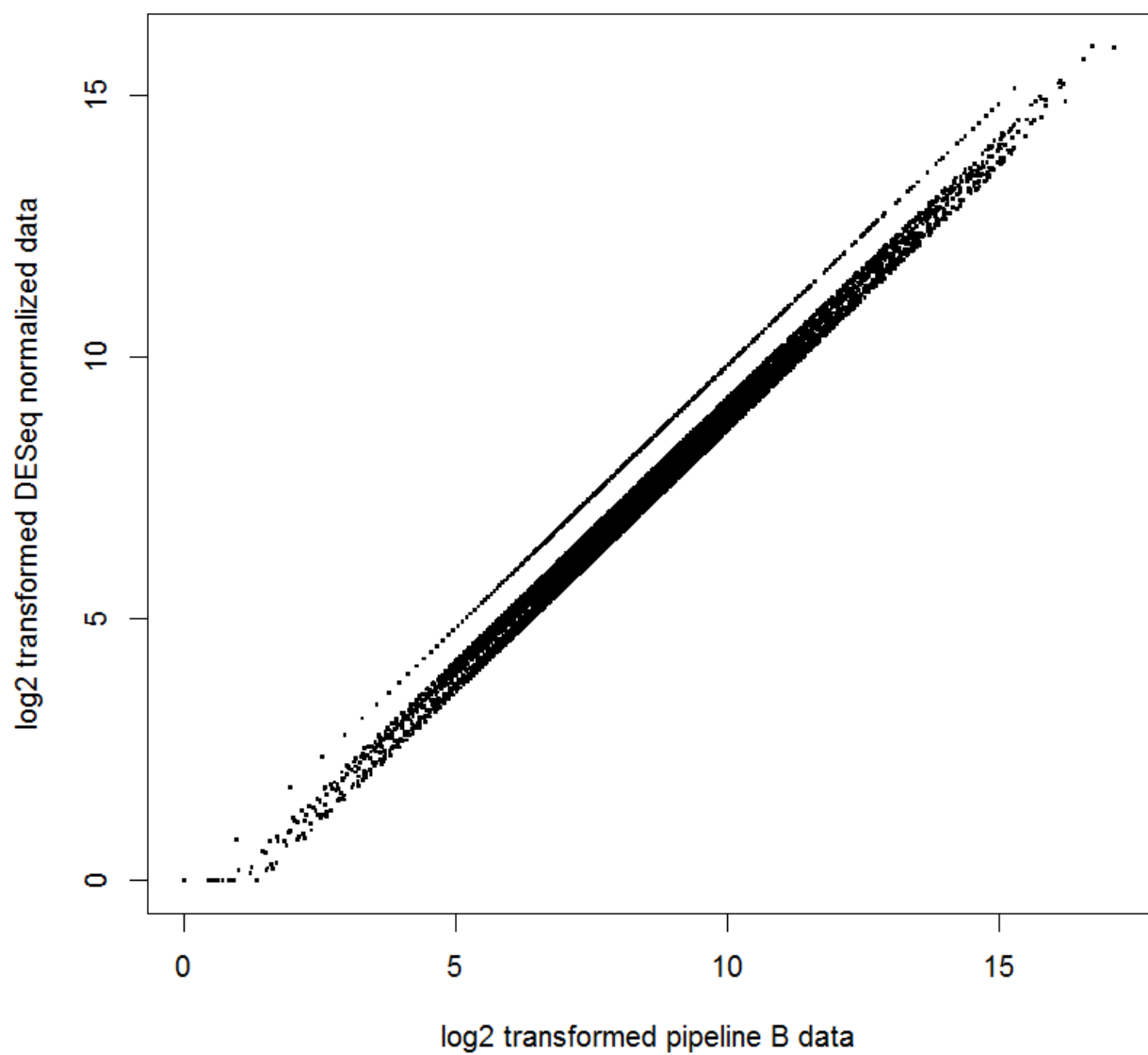

Supplement: Additional file 1 — The figure shows the comparison of DESeq and RPM normalization of Illumina high-throughput sequencing data. Each dot represents the RPM normalized (x-axis) and DESeq normalized (y-axis) HTS miRNA isoform data (pipeline B) for 1 of 20 thyroid cancer HTS data samples. Pearson correlation of data normalized independently with 2 normalizations is equal to 0.97. [file 1756-0500-7-144-S1.pdf]

A

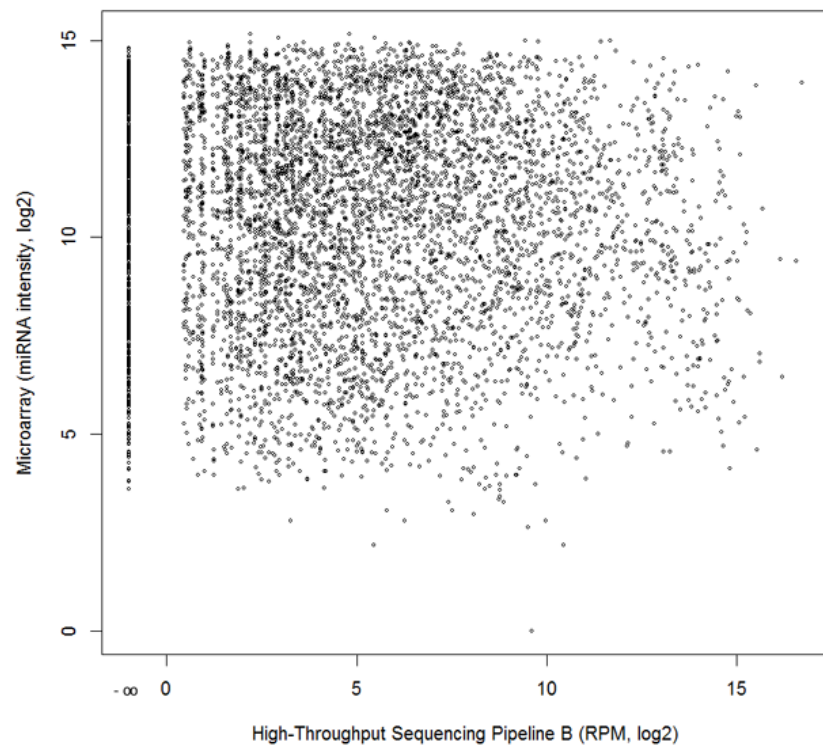

B

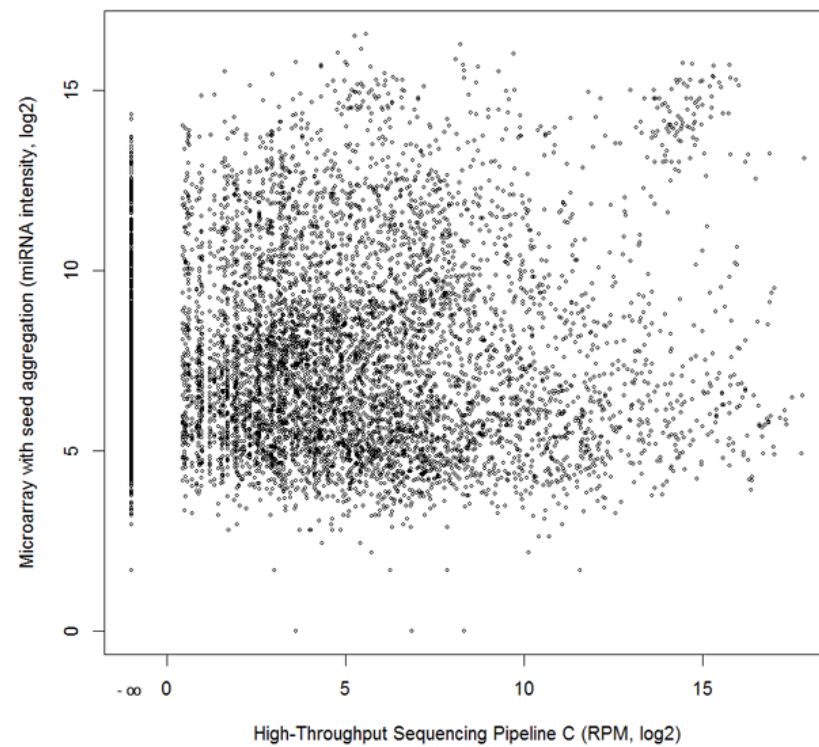

Supplement: Additional file 4 — The figure shows the comparison of Illumina high-throughput sequencing and Illumina miRNA Bead Arrays v2 performance in thyroid tumor samples. Each dot represents the sum of abundance of all individual sequence isoforms generated by pipeline B that contains the sequence of one array probe (A) or the sum of abundance of all individual sequence isoforms generated by pipeline C that share the same seed sequence (B) in 1 of 20 samples in HTS (x-axis) and the fluorescent intensity of the corresponding spot (A) or aggregation of the fluorescent intensity of corresponding spots with the same seed sequence (B) on the microarray (y-axis) of all 20 samples. Expression values are based on normalized data, assuming perfect matching of microarray probe sequence and HTS sequence reads (A) or the same seed sequence (B). The figures illustrate low correlation of microarray signals and the number of HTS reads and a lack of specificity of microarray probe hybridizations by the presence of fluorescent signal for miRNA targets without any HTS reads (x-axis position -∞). The Pearson correlation coefficient between the microarray and the HTS pipeline B or C data sets is −0.012 and 0.040, respectively. [file 1756-0500-7-144-S4.pdf]

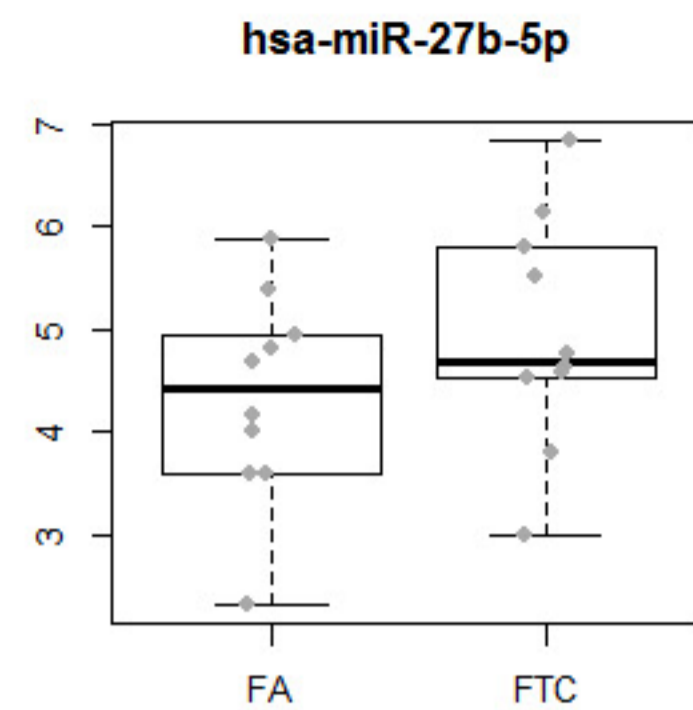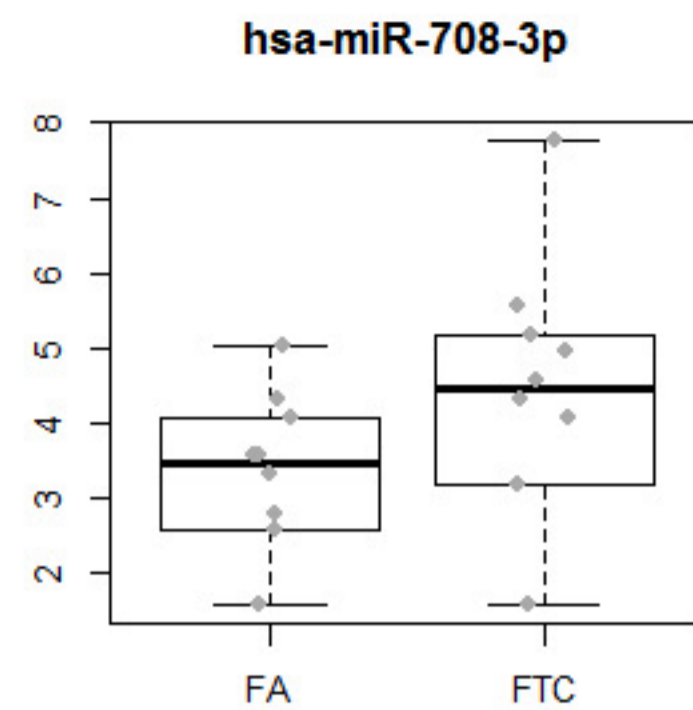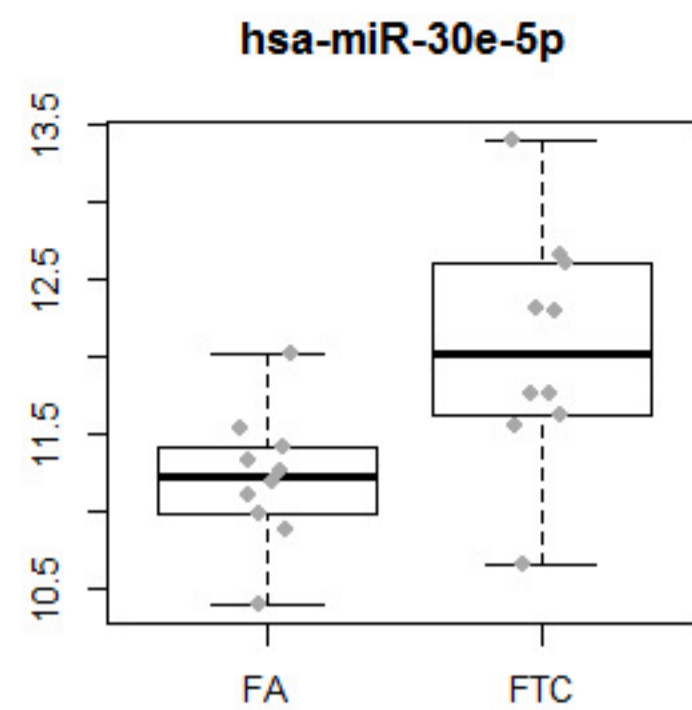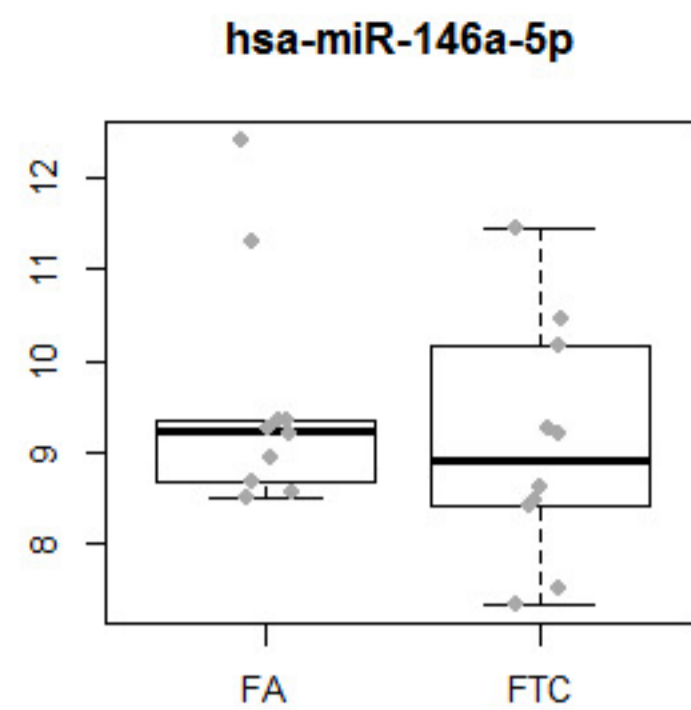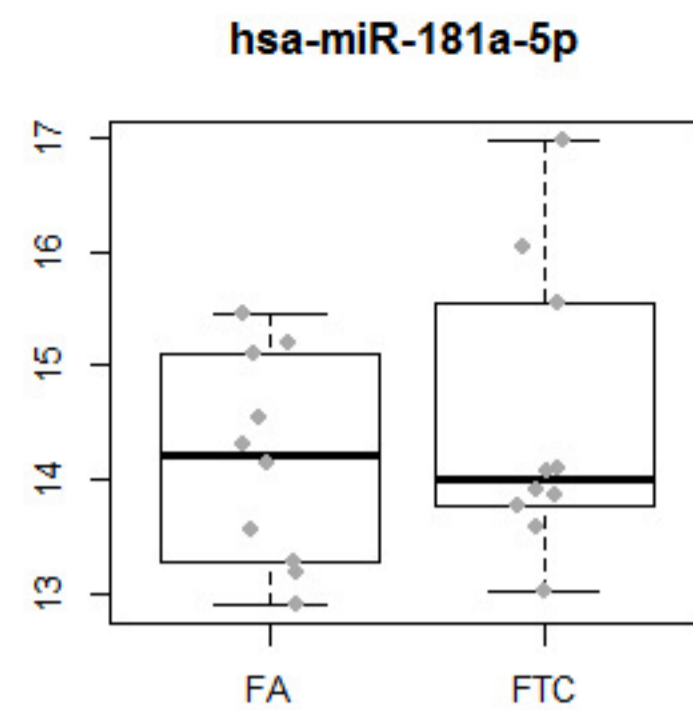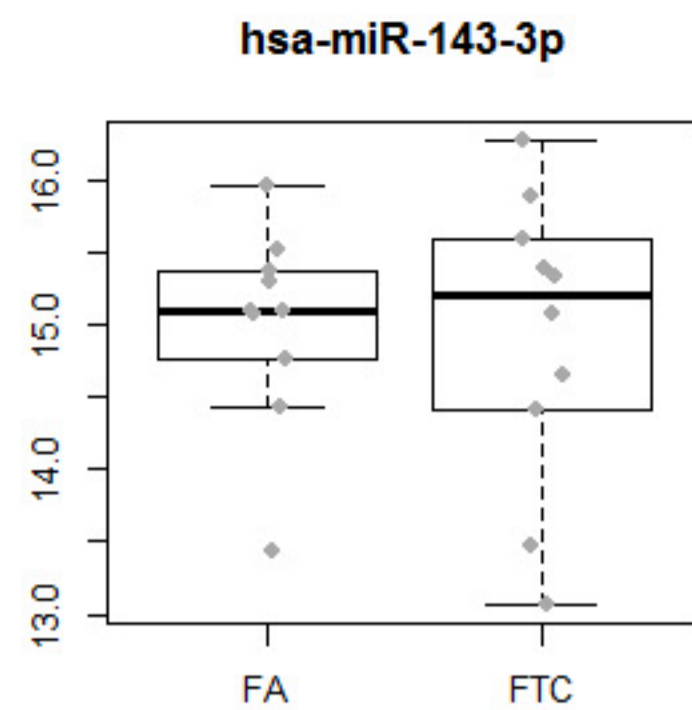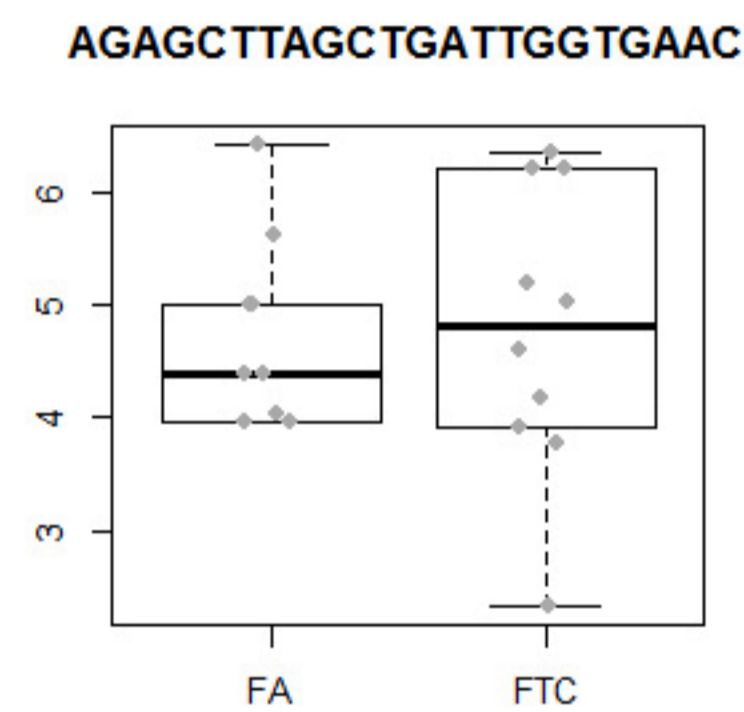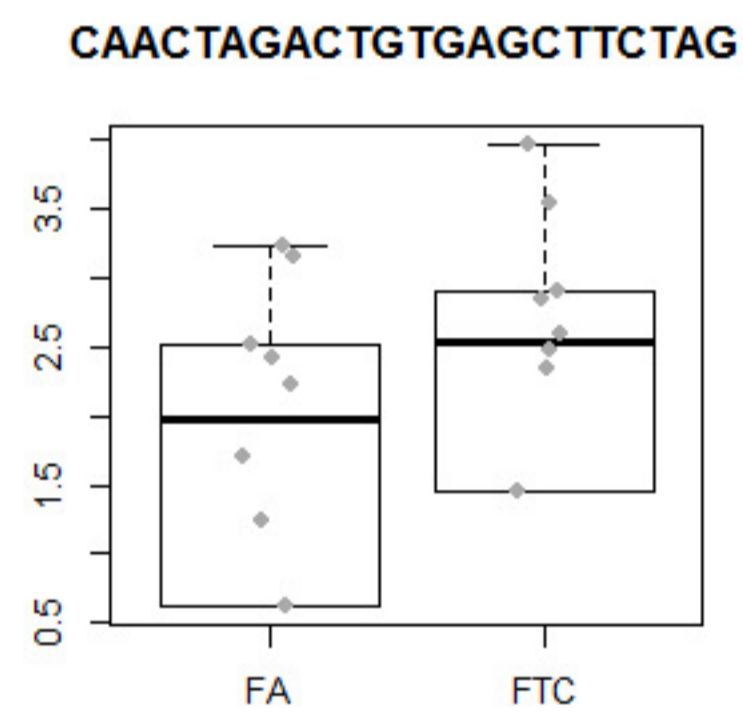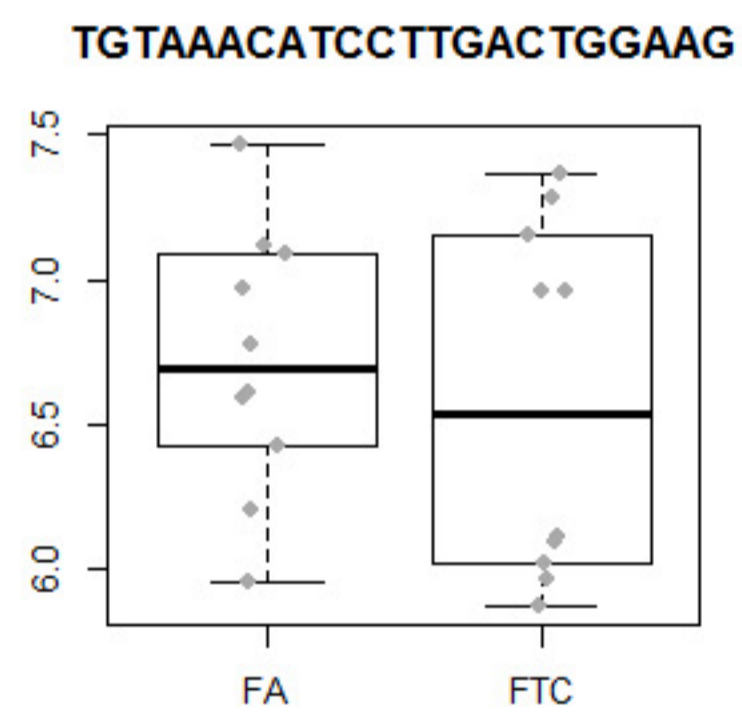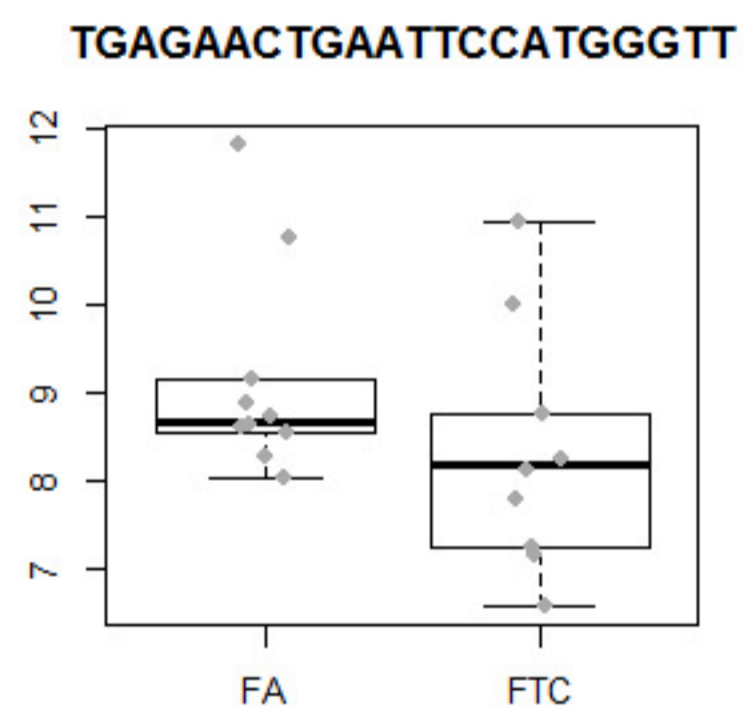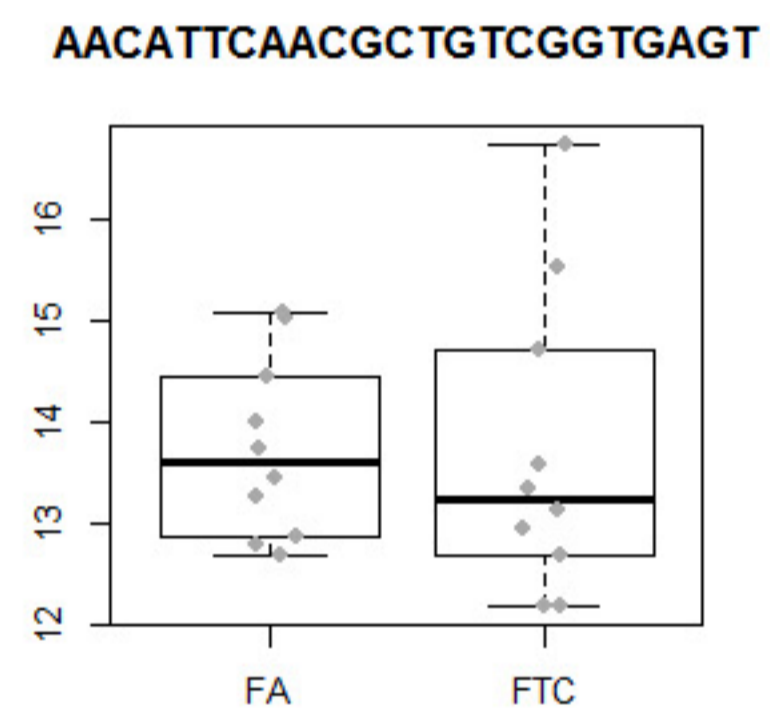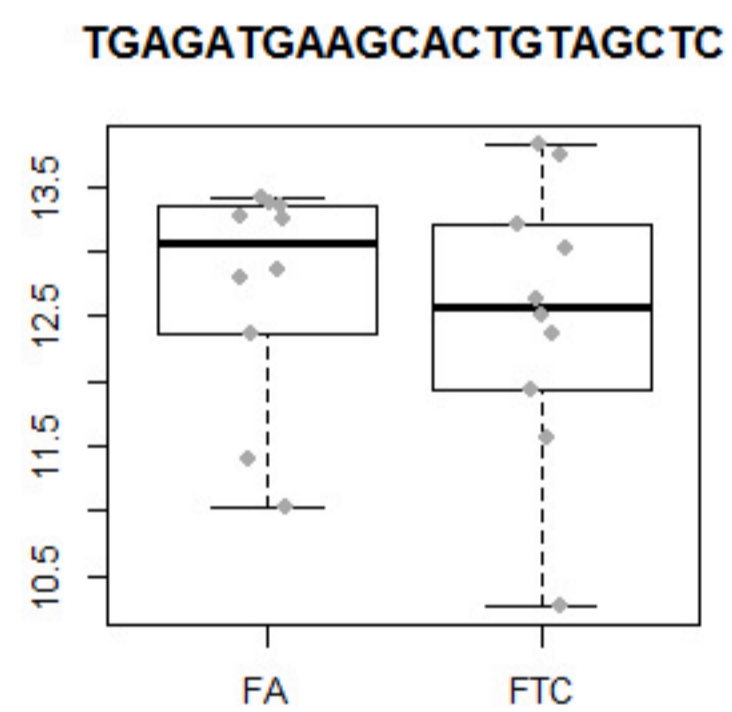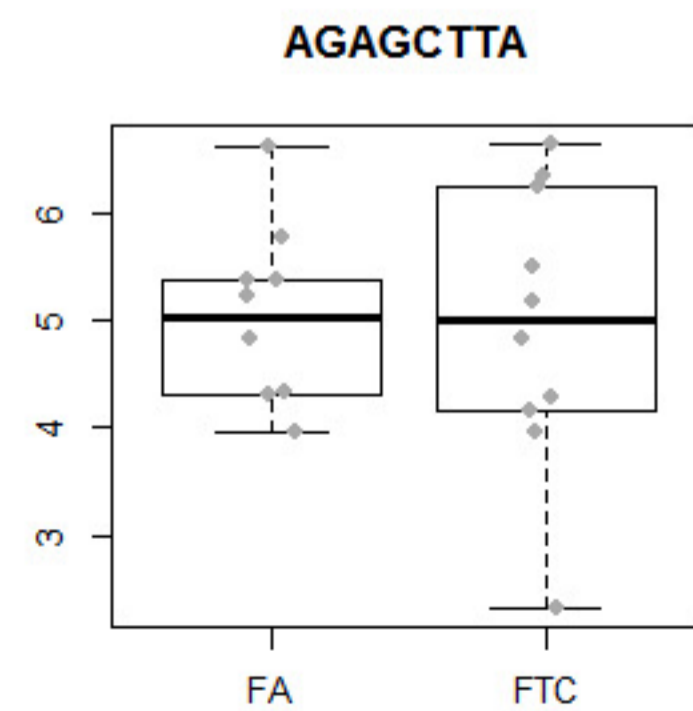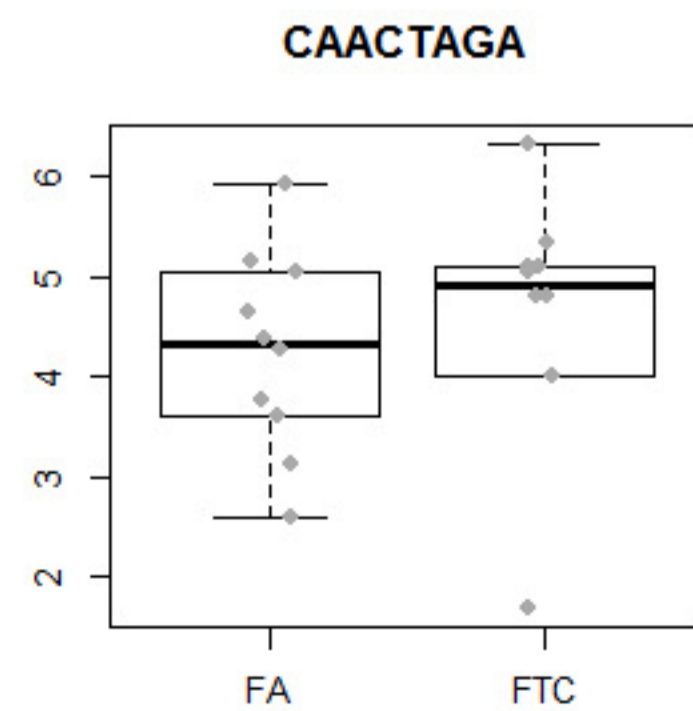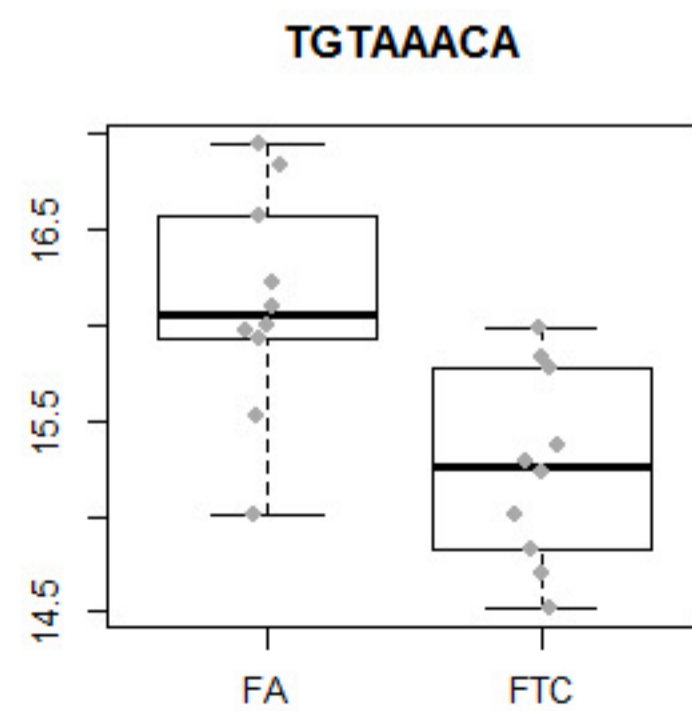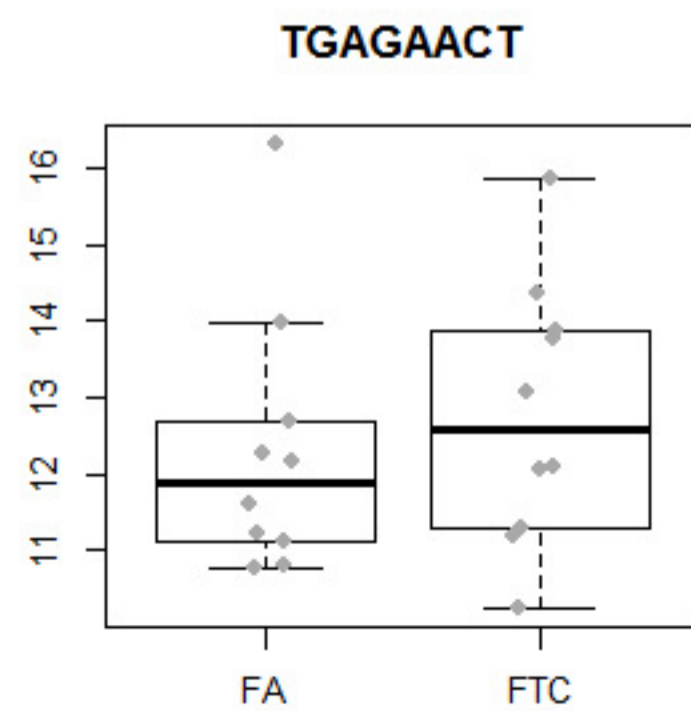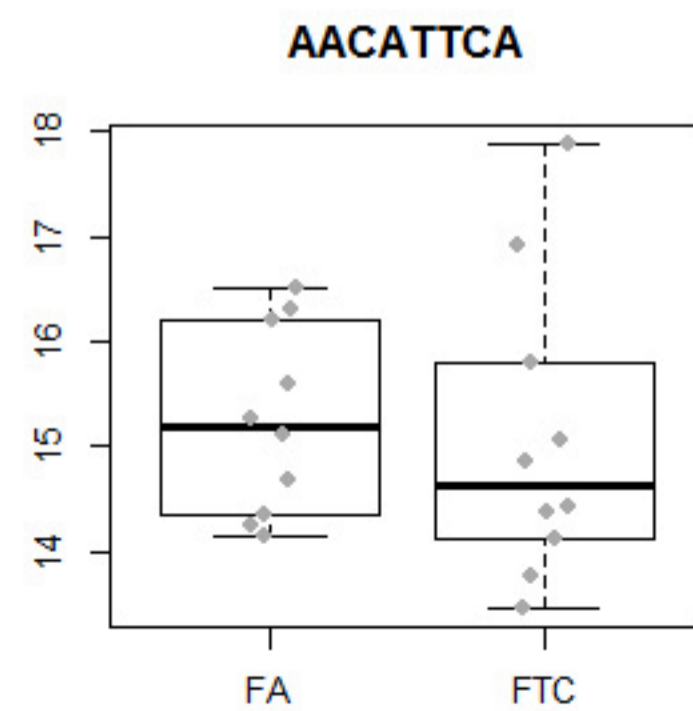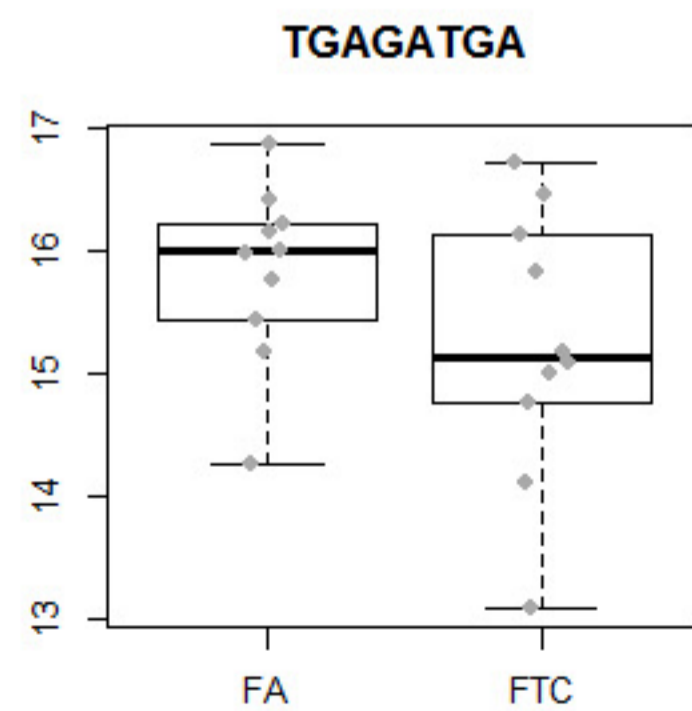

Supplement: Additional file 5 — The figure shows the expression of 6 selected miRNAs separated by tumor malignancy condition. Consecutive rows illustrate pipelines A, B and C, while box plot axis RPM normalized and log2 transformed miRNA abundance. No significant differential miRNA expression between tumor classes is observed in studied analysis pipelines. [file 1756-0500-7-144-S5.pdf]
