# Supplementary material for: Analysis options for high-throughput sequencing in miRNA expression profiling
Source: BMC Res Notes. 2014 Mar 13;7:144. doi: 10.1186/1756-0500-7-144 (PMC4007773; doi:10.1186/1756-0500-7-144)
Supplement: Additional file 2 — The table provides the qPCR outline with the respective primers. [file 1756-0500-7-144-S2.pdf]

Additional file 2. Outline of the qPCR with the respective primers.

miR target	qPCR template	Forward Primer	Reverse Primer
miR-27b	cDNA	miScript RT kit forward primer	miScript Universal primer
	HTS library	miScript RT kit forward primer	TGG CAC CCG AGA ATT CCA
miR-708	cDNA	miScript RT kit forward primer	miScript Universal primer
	HTS library	miScript RT kit forward primer	TGG CAC CCG AGA ATT CCA
miR-30e	cDNA	miScript RT kit forward primer	miScript Universal primer
	HTS library	miScript RT kit forward primer	TGG CAC CCG AGA ATT CCA
miR-146a	cDNA	miScript RT kit forward primer	miScript Universal primer
	HTS library	miScript RT kit forward primer	TGG CAC CCG AGA ATT CCA
miR-181a	cDNA	miScript RT kit forward primer	miScript Universal primer
	HTS library	miScript RT kit forward primer	TGG CAC CCG AGA ATT CCA
miR-143-1	cDNA	miScript RT kit forward primer	miScript Universal primer
	HTS library	miScript RT kit forward primer	TGG CAC CCG AGA ATT CCA
miR-30a-5p	HTS library	TGT AAA CAT CCT CGA CTG GA	CAC CCG AGA ATT CCA CT
miR-30c-5p	HTS library	TGT AAA CAT CCT ACA CTC TC	CAC CCG AGA ATT CCA CT
miR-30d-5p	HTS library	TGT AAA CAT CCC CGA CTG GA	CAC CCG AGA ATT CCA CT
miR-30e-5p	HTS library	TGT AAA CAT CCT TGA CTG GA	CAC CCG AGA ATT CCA CT
miR-30e-1	HTS library	TGT AAA CAT CCT TGA CTG GA	CCC GAG AAT TCC AGC T
miR-30e-2	HTS library	TGT AAA CAT CCT TGA CTG GA	CCC GAG AAT TCC AAG CT
