# Supplementary material for: Analysis options for high-throughput sequencing in miRNA expression profiling
Source: BMC Res Notes. 2014 Mar 13;7:144. doi: 10.1186/1756-0500-7-144 (PMC4007773; doi:10.1186/1756-0500-7-144)
Supplement: Additional file 3 — The table lists abundant uncharacterized small RNA sequences and gives the respective genomic origin. [file 1756-0500-7-144-S3.pdf]

Additional file 3. Abundant uncharacterized small RNA sequences.

Sequence	RPM mean	Locus	Start	Stop
UGUCUGAGCGUCGCU	4962	chr16	33965565	3396580
AACUUAACUUGACCGCUCUGACC	4436	chr7	145694463	145694486
UCCUGUACUGAGCUGCCCCGAGA	4066	chr8	41517962	41517985
GGGUGCUGUAGGCUU	3519	chrX	68892428	68892443
GUACGACUCUUAGCGG	3045	chr12	39770925	39770940
GCGGGUGAUGCGAACUGGAGUCUGAGC	2742	chr17	62223486	62223513
UUUCAACUUAACUUGACCGCUCUGACC	2334	chr7	145694459	145694486
CACCACGUUCCCGUGG	2198	chr7	97553704	97553720
UAGCUUCUCAGACUGAUGUUGA	2196	chr17	57918634	57918656
UGAGGUCGUAGAUUGUAUAGUU	1723	chrX	53584207	53584229
