# Supplementary material for: Analysis options for high-throughput sequencing in miRNA expression profiling
Source: BMC Res Notes. 2014 Mar 13;7:144. doi: 10.1186/1756-0500-7-144 (PMC4007773; doi:10.1186/1756-0500-7-144)
Supplement: Additional file 6 — The table lists microarray probe sequences without HTS reads but high fluorescent intensity. [file 1756-0500-7-144-S6.pdf]

Additional file 6. Microarray probe sequences without HTS reads.

Probe sequence	Fluorescent intensity (log2)
ATTCTGCATTTTTAGCAAGT	13.82
ACTTGCTGCCTTGCCACT	13.56
ACAACAGTGCCAACCTC	12.82
CCTGATTAAACACATGCTCTG	12.82
TGGCCATGGGGCTGCGCG	12.81
GCGCTTCCCTTTGCTGGA	12.37
TGATGAGCCTTGGGGAT	11.58
GGCGGGGGCAGGTGTGTG	11.53
ATCGTGCATCCCTTTAGA	11.11
TCAAATGCTCAGACTCCTG	10.87
