# Supplementary material for: Analysis options for high-throughput sequencing in miRNA expression profiling
Source: BMC Res Notes. 2014 Mar 13;7:144. doi: 10.1186/1756-0500-7-144 (PMC4007773; doi:10.1186/1756-0500-7-144)
Supplement: Additional file 7 — The table provides raw counts for miR-30a-5p, −30c-5p, −30d-5p and -30e-5p as well as for hairpin sequence of miRNA-30e (pipeline A). [file 1756-0500-7-144-S7.pdf]

Additional file 7. Raw counts for miR-30a-5p, -30c-5p, -30d-5p and -30e-5p as well as for hairpin sequence of miRNA-30e (pipeline A).

tumor sample	counts total (hg19)	hsa-miR-30a-5p	hsa-miR-30c-5p	hsa-miR-30d-5p	hsa-miR-30e-5p	hsa-mir-30e
FA1	4001179	170036	7280	20153	9308	12341
FA2	1731162	46062	2818	6170	2334	3457
FA3	490090	7179	895	2998	1081	1398
FA4	263447	15223	955	2237	1091	1733
FA5	190791	2382	190	790	358	449
FA7	1155487	32579	4100	8747	3430	4263
FA8	1065008	27677	2418	4633	2609	3442
FA9	1291111	72524	4258	11839	3512	4302
FA10	1022666	27757	2909	8389	2647	3323
FA11	1064393	28732	2748	7366	2153	2875
FTC2	372384	3378	361	3111	4001	5421
FTC3	1237471	13289	1338	3135	1986	2756
FTC5	966616	20887	1446	7032	5987	7481
FTC6	393996	3592	926	6728	1968	2360
FTC7	1315541	34530	2668	10253	4539	5811
FTC9	559439	12018	1295	6170	2850	3534
FTC12	550354	10287	796	5494	1734	2374
FTC14	291370	8247	279	2165	1881	2261
FTC15	1077727	30161	2931	9361	3223	4009
FTC16	802332	7812	907	3556	2774	3329
